# Glycoside Hydrolases and Non-Enzymatic Glycation Inhibitory Potential of *Viburnum opulus* L. Fruit—In Vitro Studies

**DOI:** 10.3390/antiox10060989

**Published:** 2021-06-21

**Authors:** Dominika Kajszczak, Agnieszka Kowalska-Baron, Anna Podsędek

**Affiliations:** 1 Institute of Molecular and Industrial Biotechnology, Faculty of Biotechnology and Food Sciences, Lodz University of Technology, Stefanowskiego 2/22, 90-537 Łódź, Poland; anna.podsedek@p.lodz.pl; 2 Institute of Natural Products and Cosmetics, Faculty of Biotechnology and Food Sciences, Lodz University of Technology, Stefanowskiego 2/22, 90-537 Łódź, Poland; agnieszka.kowalska-baron@p.lodz.pl

**Keywords:** *V. opulus* fruit, α-amylase, α-glucosidase, glycation end-products, antioxidant capacity, phenolic compounds

## Abstract

Phytochemicals of various origins are of great interest for their antidiabetic potential. In the present study, the inhibitory effects against carbohydrate digestive enzymes and non-enzymatic glycation, antioxidant capacity, and phenolic compounds composition of *Viburnum opulus* L. fruits have been studied. Crude extract (CE), purified extract (PE), and ethyl acetate (PEAF) and water (PEWF) fractions of PE were used in enzymatic assays to evaluate their inhibitory potential against α-amylase with potato and rice starch as substrate, α-glucosidase using maltose and sucrose as substrate, the antioxidant capacity (ABTS, ORAC and FRAP assays), antiglycation (BSA-fructose and BSA-glucose model) properties. Among four tested samples, PEAF not only had the highest content of total phenolics, but also possessed the strongest α-glucosidase inhibition, antiglycation and antioxidant activities. UPLC analysis revealed that this fraction contained mainly chlorogenic acid, proanthocyanidin oligomers and flavalignans. Contrary, the anti-amylase activity of *V. opulus* fruits probably occurs due to the presence of proanthocyanidin polymers and chlorogenic acids, especially dicaffeoylquinic acids present in PEWF. All *V. opulus* samples have an uncompetitive and mixed type inhibition against α-amylase and α-glucosidase, respectively. Considering strong anti-glucosidase, antioxidant and antiglycation activities, *V. opulus* fruits may find promising applications in nutraceuticals and functional foods with antidiabetic activity.

## 1. Introduction

Type 2 diabetes mellitus (T2DM) is a global problem because it concerns hundreds millions of people and has a significant impact on the health, quality of life and the health care system [1]. Evidence from epidemiological studies suggests that many cases of T2DM can be prevented by improving the main modifiable risk factors such as obesity, sedentary lifestyle and unhealthy diet contains large amounts of carbohydrates and fats [2,3]. Therefore, diet, weight control and exercise are essential and effective means of improving glucose homeostasis. Some medicinal plants, fruits, and vegetables have been reported in the literature as having been used to control diabetes [4,5,6,7,8,9]. In addition, the antidiabetic agents from the natural resources can be an alternative to hypoglycemic drugs that are not always satisfactory and show side effects [10,11]. The antidiabetic activity of plant-derived foods and extracts has been attributed, inter alia, to the naturally available phenolic compounds [6,12,13,14,15,16,17,18,19]. These secondary plant metabolites comprise one (phenolic acids such as hydroxybenzoic and hydroxycinnamic acids) or more (polyphenols containing flavonoids, tannins, lignans) aromatic rings with attached hydroxyl group in their structures [20]. These secondary plant metabolites have the capacity to inhibit activity of carbohydrate hydrolyzing digestive enzymes, modulate the glucose transport, stimulate insulin secretion from pancreatic *β*-cells, reduce insulin resistance, increase insulin sensitivity, inhibit the activity of protein tyrosine phosphatase, reduce the production of protein glycation products, and improve antioxidant defenses [15,21,22,23]. The listed activities of phenolic compounds largely depend on their structure [24,25,26,27]. For example, the hydroxylation on aromatic rings of flavonoids improved the inhibition against α-amylase and α-glucosidase while the glycosylation of hydroxyl group on flavonoids weakened the inhibitory potential [24]. Generally, the glycosylation and methylation/methoxylation of flavonoids decrease the antiglycation effects in vitro models [28]. In addition, the antiglycation activities of flavones were stronger than those of flavonols, flavanones or isoflavones [29].

A promising candidate capable of attenuating T2DM might be phytochemicals present in different morphological parts of the monotypic family Viburnaceae. Supplementation of ethanol extract of *Viburnum stellato-tomentosum* aerial parts at 150 mg/kg dose one a day for 17 weeks significantly decreased fasting glucose insulin, homeostasis model assessment of insulin resistance in high-fat diet fed C57BL/6J mice [30]. The freeze-dried powder of *V. dilatum* fruit juice orally administered to streptozotocin-induced diabetes rats at 500 mg/kg dose significantly decreased plasma glucose level without increase of insulin secretion [31]. However, studies with the Swiss albino mice with alloxane-induced diabetes showed that water extract obtained from *V. opulus* leaves at 100 mg/kg dose had no effect in terms of lowering blood glucose [32]. Our in vitro cell-based study demonstrated that phenolic rich fraction obtained from *V. opulus* fruit juice and methanol-acetone extract from the remaining pomace decreased the uptake of fluorescent glucose analogue 2-(N-(7-nitrobenz-2-oxa-1,3-diazol-4-yl)amino)-2-deoxyglucose by human adenocarcinoma Caco-2 cells [33]. The next study demonstrated that *V. opulus* fruit juice and juice enriched with phenolics decreased glucose-stimulated insulin secretion in the mouse insulinoma cell line MIN6 and increased insulin secretion at low glucose concentrations [34]. Furthermore, both juices intensified free fatty acid uptake and lipid accumulation in MIN6 cells cultured in the presence of an elevated concentration of palmitic acid. Additionally, a screening study with a cell-free assay identified *V. opulus* fruit pulp acetone extract as inhibitors of carbohydrates hydrolyzing enzymes (α-amylase and α -glucosidase) or protein tyrosine phosphatase, which is known as the major negative regulator in insulin signaling [35]. In addition to antidiabetic activity, in vitro studies have demonstrated anti-obesity, anti-inflammatory, anti-cancer, and antimicrobial effects of *V. opulus* fruits. In animal studies, a beneficial effect on the urinary system and vasorelaxant activities were also demonstrated [36]. *V. opulus* is common in natural habitats on the European continent and in some regions of North Africa and North Asia, and also in the central zone of Russia. It is known by many names, like the Guelder rose, European guelder, European cranberrybush, rose elder, Rose Ebru, and as gilaburu in Turkey [36]. *V. opulus* fruits have been suggested as a rich source of phenolic compounds, including phenolic acids, proanthocyanidins, anthocyanins, flavonols, and flavalignans [36,37].

The aim of the presented research was to determine the antidiabetic activity of *V. opulus* fruit components by determining the effect on: (1) α-amylase activity in the presence of potato and rice starch; (2) α-glucosidase activity with maltose and sucrose as substrate; (3) the formation of glycation end products (AGE) in the BSA-fructose and BSA-glucose model, and also to estimation the antioxidant capacity using ABTS, FRAP and ORAC methods. Especially the objective of this study was to establish which phenolic compounds are responsible for the tested properties. For this purpose, the four samples were obtained such as the crude acetone extract (CE), purified extract (PE), and also water and ethyl acetate fractions obtained from PE, and their phenolic profiles were identified by the UPLC/MS method.

## 2. Materials and Methods

### 2.1. Standards and Reagents

Intestinal acetone powder from rat sources of α-glucosidase, α-amylase from porcine pancreas type VI-B, TRIS-base, D-glucose, D-fructose, aminoguanidine hydrochloride, sodium azide, bovine serum albumin (BSA), acarbose, caffeic acid, (+)-catechin, cinchonine, starch from rice, sodium chloride, maltose, sucrose, formic acid, phosphate buffered saline, 2,2′- azobis(2-amidinopropane) dihydrochloride (AAPH), 2,2′-azinobis(3-ethyl-benzthiazoline-6-sulphonic acid) (ABTS), fluorescein, 6-hydroxy-2,5,7,8-tetramethychroman-2-carboxylic acid (Trolox), iron(III) chloride, potassium persulfate, 2,4,6-tris-2-pyridyl-s-triazine (TPTZ), and acetonitrile were obtained from Sigma Aldrich (Steinheim, Germany). Acetone, methanol, ethyl acetate, hydrochloric acid, iodine, potassium iodide, disodium phosphate and monosodium phosphate were purchased from Chempur (Piekary Śląskie, Poland). Potato starch and calcium chloride were purchased from POCH (Gliwice, Poland). Chlorogenic acid, cyanidin, quercetin 3-glucoside, quercetin 3-rutinoside, and quercetin 3-rhamnoside were obtained from Extrasynthese (Lyon, France). Neochlorogenic acid, cryptochlorogenic acid, procyanidin C1 were purchased from PhytoLab (Vestenbergsgreuth, Germany). Ultrapurity water was prepared in the laboratory using a Simplicity Water Purification System (Millipore, Marlborough, MA, USA).

### 2.2. Plant Material and Samples Preparation

**Figure 1:**
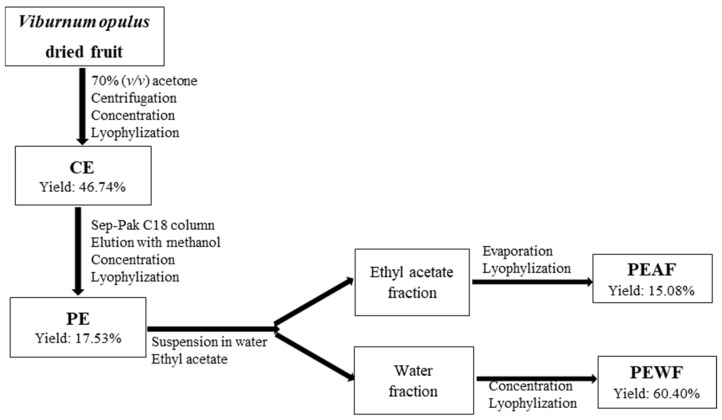
Separation strategy used to obtain the four samples of *V. opulus* fruits. CE—crude extract; PE—purified extract; PEAF—ethyl acetate fraction of purified extract; PEWF—water fraction of purified extract.

The dried fruits of *V. opulus* were purchased from “Natura Wita Ltd.” (Kopernia, Poland; https://natura-wita.pl/kontakt.html, accessed on 15 June 2021). Before the extraction process, dry fruits were ground in a coffee grinder and extracted with 70% acetone (1:20, *w*/*v*) on a magnetic stirrer at room temperature for 3 h. Then, the mixture was incubated at room temperature for 18 h followed by the extraction on a magnetic stirrer at room temperature for 3 h. After centrifugation at 5000 rpm for 10 min the supernatant was evaporated at 40 °C under reduced pressure in order to remove acetone and lyophilized to obtain the crude extract (CE). The part of CE was applied onto a Sep-Pak C18 cartridge (10g capacity, Waters Corp., Milford, MA, USA) that was previously activated with methanol and water. The column was washed with water in order to eliminate carbohydrates, proteins and other polar compounds. The phenolic compounds were then eluted with methanol. Methanol fraction was evaporated under reduced pressure (T < 40 °C), and the aqueous phase was lyophilised to afford purified extract (PE). The part of PE (300 mg) was suspended in 30 mL of water, and then, partitioned with 30 mL of ethyl acetate, repeated three times. The ethyl acetate fraction was evaporated to dryness, solubilized with water and lyophilised. This sample was named ethyl acetate fraction of purified extract (PEAF). Water fraction was concentrated, lyophilised and called water fraction of purified extract (PEWF). The yield and abbreviations for each of *V. opulus* samples are shown in Figure 1. For in vitro analysis CE, PE, PEWF and PEAF were dissolved in water at a concentration 10 mg/mL.

### 2.3. Quantification of Individual Phenolic Compounds

UPLC analysis was performed on an ultra-performance liquid chromatograph (Waters Acquity UPLC system, Milford, MA, USA) equipped with a binary pump, an autosampler, a column compartment and a diode array detector. Briefly, samples were eluted with a gradient of solvent A (4.5% formic acid in ultrapure water) and B (acetonitrile) on an Acquity UPLC HSS T3 C18 column (150 × 2.1 mm, 1.8 µm; Waters) operating at 30 °C as described in the previous work [38]. The gradient program was as follows: initial conditions 99% (A), 12 min 75% (A), 12.5 min 100% (B), 15.0 min 99% (A). The flow rate was 0.45 mL/min and the injection volume was 5 µL. The identification of phenolic compounds was described in detail previously [39]. The content of neochlorogenic acid, chlorogenic acid, cryptochlorogenic acid, caffeic acid, procyanidin C1, quercetin 3-glucoside, quercetin 3-rutinoside, and quercetin 3-rhamnoside were quantified using corresponding standard calibration curves. A quantificative analysis of other phenolics was based on the standards as follow: chlorogenic acid was used for the hydroxycinnamic acid derivatives, (+)-catechin for (epi)-catechin hexoside, procyanidin C1 for procyanidin tetramer, cinchonine for cinchonain Ix derivatives, quercetin 3-glucoside for quercetin 3-sambubioside. The results were expressed as mg/g CE, PE, PEWF, and PEAF.

### 2.4. Total Proanthocyanidins Content

The content of total proanthocyanidins was determined after their acid depolymerization to the corresponding anthocyanidins as described by Rösch et al. [40] and calculated by the molar extinction coefficient of cyanidin (ε = 17,360 L/mol^−1^*·*cm^−1^ and molar mass 287 g/mol), and was expressed as mg of cyanidin equivalents (CYE)/g of *V. opulus* samples.

### 2.5. α-Amylase Inhibition Assay

The α-amylase inhibition assay was based on a previously-described spectrophotometric method [41] with some modifications. All reagents were prepared with 0.1 M phosphate buffer containing 6 mM CaCl_2_ (pH 6.9). Briefly, 20 µL of diluted *V. opulus* samples and 40 µL of potato starch (0.83 g/L) or starch from rice (1 g/L) solution were mixed with 20 µL of α-amylase (0.1 mg/mL for potato starch and 0.2 mg/mL for starch from rice) in a 96-well plate. After incubation at 37 °C for 10 min, the reaction was stopped by addition of 80 µL of 0.4 M HCl followed by 100 µL of 5 mM I_2_ in 5 mM KI. The absorbance was read at 600 nm using a microplate reader (Synergy2, Biotek Instruments Inc., Winooski, VT, USA). Acarbose and chlorogenic acid were also used as a positive control and reference phenolic compound, respectively. Each sample was measured in triplicate. The IC_50_ value was calculated by regression analysis as the mean concentration of tested sample required to demonstrating at 50% inhibition against α-amylase in this condition.

### 2.6. α-Glucosidase Inhibition Assay

The assessment of the α-glucosidase activity was slightly modified according to Adisakwattana et al. [42] and Gao et al. [43]. Briefly, 125 mg of rat intestinal acetone powder was mixed with 2.5 mL of 0.9% NaCl solution. Enzyme isolation was performed in an ultrasonic cleaner (for 30 sec) in an ice bath, then 30 sec without ultrasonic. This step was repeated 12 times. After that, the solution was centrifuged at 4 °C (3000 rpm, 30 min). 50 μL of diluted *V. opulus* samples were mixed with 50 μL of enzyme supernatant (diluted 2 times for reaction with maltose). After incubation at 37 °C for 10 min, 50 μL of substrate (0.1 M maltose or 0.5 M sucrose solution prepared in 0.1 M phosphate buffer, pH 6.9) was added and incubated in a 96-well microplate at 37 °C for 20 min for maltose and 60 min for sucrose. The reaction was stopped by adding 150 µL of 2 M Tris-HCl buffer (pH 7.0). The concentration of glucose released from the reaction mixture was determined by the commercial glucose test (BIOMAXIMA SA, Lublin, Poland). Acarbose and chlorogenic acid were also used as a positive control and reference phenolic compound, respectively. The concentration of the tested sample able to inhibit the α-glucosidase by 50% (IC_50_ value) was calculated by regression analysis.

### 2.7. Mode of Enzyme Inhibition

The α-amylase and α-glucosidase inhibition modes of different *V. opulus* fruit samples (PE, PEWF, PEAF) and reference substances (chlorogenic acid and acarbose) were determined by Lineweaver-Burk model. The enzymes activities without and with tested samples were studied using the methods described in Section 2.5 and Section 2.6. A double reciprocal graph (1/v versus 1/S, where “v” is the reaction rate and “S” the concentration of the substrate), was plotted. All samples were tested at three different doses using six concentrations of substrate.

### 2.8. Fluorescence Measurements

The effect of PE on fluorescence spectra of α-amylase and α-glucosidase at different concentrations of PE (from 0.014 to 0.136 mM) were performed using FluoroMax 4 (Jobin Yvon Spex, UK, Stanmore, Middlesex) spectrofluorometer (Horiba Scientific, Piscataway, NJ, USA) using a method described by Zhu et al. [44]. The fluorescence spectra of enzymes and their changes upon adding increasing amounts of PE of *V. opulus* fruits were recorded in the wavelength range of 315–450 nm upon excitation at 295 nm. Both excitation and emission slit widths were set at 2 nm. All measurements were performed in a standard quartz cuvette at 20 °C. A 2.5 mL solution containing 2 × 10^−6^ M α-amylase and 1.3 × 10^−6^ M α-glucosidase in 0.01 M PBS (pH 7.4), was titrated by successive additions of PE to give a final concentration 1.36 × 10^−4^ M. The PE concentrations were expressed as chlorogenic acid (M = 354.31 g/mol) equivalents.

The time-resolved fluorescence of α-amylase and α-glucosidase before and after adding increasing concentrations of PE was measured using the FL900CDT time-correlated single-photon counting fluorimeter (Edinburgh Analytical Instruments, Edinburgh, UK). The excitation and emission wavelengths were set to 295 and 350 nm, respectively. The instrumental response function was ~1.18 ns (FWHM full width at half-maximum). The time-resolved measurements were performed at 20 °C in a standard quartz cuvette. Data acquisition and analysis of the obtained results were performed with the use of software provided by Edinburgh Analytical Instrumentation (F900). The goodness of fit was estimated by using χ2 values.

### 2.9. Protein Glycation Inhibition Assay

The bovine serum albumin (BSA) and glucose/fructose assay was carried according to the method of Błaszczak et al. [45] with some modifications. We prepared 25 mL of the test solution containing D-glucose or D-fructose (1.0 M), BSA (10 mg/mL) and sodium azide (0.1 mg/mL) in the phosphate buffer (0.1 M, pH 7.4). The test solution (1 mL) was incubated in the dark at 37 °C for 13 days for glucose and for 6 days for fructose with or without 0.5 mL of tested *V. opulus* samples. Aminoguanidine was used as positive control in the concentration from 41.67 to 166.67 μg/mL for glucose and from 22.22 to 133.33 μg/mL for fructose. Next, the formation of AGE was evaluated based on fluorescence intensity at λ = 330 nm (excitation wave) and λ = 460 nm (emission wave). Fluorescence intensity was measured in 96-well plates (black, OptiPlate-96 F, PerkinElmer, UK) in a microplate reader (Synergy2, BioTek Instruments Inc., Winooski, VT, USA).

### 2.10. In Vitro Antioxidant Assays

Antioxidant capacity of *V. opulus* fruit samples was determined by ABTS radical cation (ABTS), oxygen radical (ORAC) scavenging capacity, and as ferric reducing power (FRAP). Antioxidant capacity was expressed as mM Trolox equivalents (TE) per g of CE and PE extracts, and PEAF and PEWF fractions. ABTS and FRAP assays procedures have been detailed in our previous works [41,46]. The ABTS^•+^ monocation was generated by oxidation of ABTS with potassium persulphate. The tested sample (20 µL) was added to the ABTS^•+^ solution (1 mL) and after 6 min, the absorbance at 734 nm was measured. In the FRAP assay, 90 µL of tested sample was mixed with 0.27 mL of water and freshly prepared FRAP reagent (2.7 mL). After 10 min the absorbance at 593 nm was measured. ORAC assay was carried out using a Synergy^TM^2 microplate reader (BioTek Instruments Inc., Winooski, VT, USA). Peroxyl radicals were generated by the spontaneous decomposition of AAPH at 37 °C. The loss of fluorescence of fluorescein was an indication of the extent of damage from its reaction with the peroxyl radicals. Fluorescence was read at 485 nm excitation and 520 nm emission every 2 min for 2 h.

### 2.11. Statistical Analysis

All samples were assayed in triplicate and results are given as the mean ± standard deviation using Microsoft Excel XP. Significance differences were calculated using one-way analysis of variance (ANOVA) using Statistica Ver. 6.0 (TIBCO Software Inc., Palo Alto, CA, USA) followed by post hoc Tukey test. Statistically significant differences were set at *p* < 0.05.

## 3. Results

### 3.1. Effects of V. opulus Fruit Components on α-Amylase and α-Glucosidase Activity

The inhibitory effects of different samples separated from *V. opulus* dried fruits such as crude extract (CE), purified extract (PE), and two fractions separated from PE, called water fraction (PEWF) and ethyl acetate fraction (PEAF), were analyzed by the dose-effect plots (Supplementary Figure S1). In the present study, the α-amylase assay was performed in two systems using potato starch or starch from rice as substrates. Measurement of α-glucosidase activity was also carried out in two systems using sucrose or maltose as substrates. Inhibitory activity of extracts and fractions of *V. opulus* dried fruit had a direct linear relationship between concentration and percentage inhibitory activity.

The degree of inhibition of the tested enzymes depended on both the type of *V. opulus* sample and the type of substrate used. The *V. opulus* samples differed significantly (*p* < 0.05) in their IC_50_ values (Table 1). PEWF was the strongest inhibitor of porcine pancreatic α-amylase while PEAF against α-glucosidase as indicated by the lowest IC_50_ values. CE and PEAF performed a weak inhibitory effect on α-amylase while CE on α-glucosidase. Purification of CE by solid-phase extraction on the C18 bed increased the inhibitory activity of PE against α-amylase 6.5-fold and almost 4-fold using potato starch or starch from rice, respectively. In the case of α-glucosidase, the inhibitory activity of PE exceeded almost 11-fold and more than 7-fold the effectiveness of CE in the assay system containing maltose or sucrose as a substrate, respectively. However, the comparison of the IC_50_ values for PEWF and PEAF, i.e., fractions separated from PE by liquid-liquid extraction with ethyl acetate, indicates a higher affinity of α-amylase inhibitors to water, and α-glucosidase inhibitors to ethyl acetate. All *V. opulus* fruit samples showed lower inhibitory activity against both enzymes compared to acarbose, with greater differences occurring with α-glucosidase. Chlorogenic acid—the reference phenolic compound—was a weaker inhibitor of both glycoside hydrolases compared to acarbose, and the weaker α-amylase inhibitor compared to all *V. opulus* fruit samples. In contrast, in the case of α-glucosidase, chlorogenic acid showed comparable inhibition efficiency to PEAF in the assay system with sucrose, but exhibited 1.7-fold lower inhibitory activity than PEAF in the system using maltose as a substrate.

A Lineweaver-Burk plot between 1/[substrate] and 1/V (reaction rate) was used to examine the action type of the most active *V. opulus* samples (PE and PEWF) against α-amylase (Supplementary Figure S2), or PE and PEAF samples against α-glucosidase (Supplementary Figure S3). For comparison, chlorogenic acid and acarbose were also tested.

In the study, the kinetic Michaelis-Menten parameters such as Michaelis constant (Km), maximum rate (Vmax), and inhibition constant (Ki) were calculated from Lineweaver-Burk graphs (Supplementary Figures S2 and S3) and are summarized in Table 2. All tested *V. opulus* samples similar to acarbose have an uncompetitive type inhibition against α-amylase while chlorogenic acid inhibits this enzyme activity in competitive manner, regardless of the type of starch. Whereas, in the case of α-glucosidase, the tested *V. opulus* samples and acarbose have a mixed type of inhibition (competitive and noncompetitive), regardless of the type of disaccharide as substrate. The results also suggest that chlorogenic acid has a noncompetitive-type inhibition against this enzyme in both assay models.

### 3.2. Effect of PE on α-Amylase and α-Glucosidase Spectra

**Figure 2:**
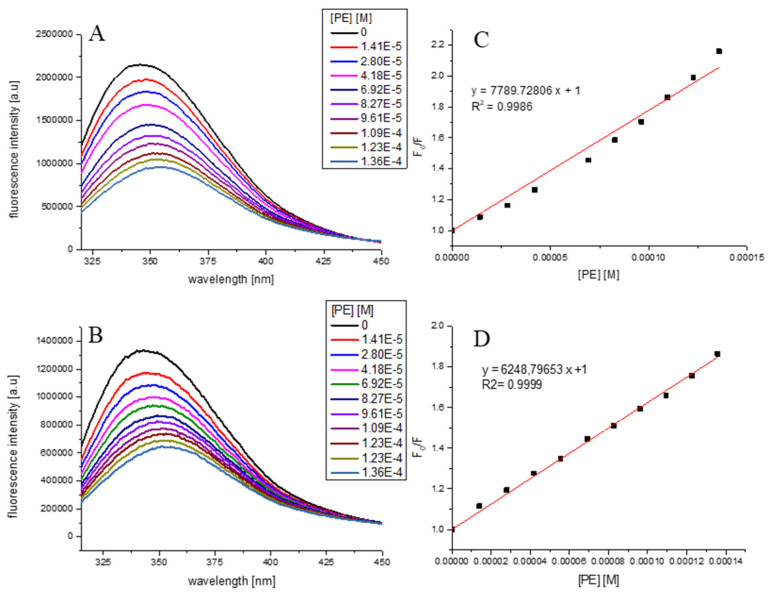
Fluorescence quenching spectra of α-amylase (**A**) and α-glucosidase (**B**) at λ_ex_ = 295 nm. PE concentration increased from 0 to 1.36 × 10^−4^ M. The Stern–Volmer plots for α-amylase (**C**) and α-glucosidase (**D**) fluorescence quenching by PE.s.

The fluorescence spectra of α-amylase and α-glucosidase at different concentrations of PE (0.014–0.136 mM as chlorogenic acid equivalents) are depicted in Figure 2. It was evident that with the increasing concentration of PE, the fluorescence intensity of both enzymes reduced progressively, which is indicative of molecular interactions between PE components and α-amylase as well as α-glucosidase. From Figure 2A,B it can be seen that addition of PE to α-amylase or α-glucosidase solution resulted in tryptophan fluorescence quenching. Moreover, a slight redshift of the tested enzymes emission spectra maximum may be observed. Due to the presence of this alteration to the fluorescence maximum, the Stern–Volmer plots (Figure 2C,D) were obtained from the integrated fluorescence intensities (the area under the spectrum with the wavelength range of 310–450 nm). The determined value of the Stern–Volmer constant was 7.790 ± 0.226 × 10^3^/M for α-amylase and 6.249 ± 0.060 × 10^3^/M for α-glucosidase. The results of time-resolved fluorescence measurements (Table 3) have indicated that the average lifetimes (<τ>) of α-amylase and α-glucosidase (<τ> of around 12 and 11 ns, respectively) have not changed significantly upon adding PE up to the concentration 1.36 × 10^−4^ M. This observation may indicate that PE quenches the tryptophan fluorescence of α-amylase or α-glucosidase via a static quenching mechanism.

The bimolecular quenching constant of tryptophan α-amylase or α-glucosidase fluorescence by PE was determined from the following equation

(1) $$\frac{F_{0}}{F}=1+k_{q}\langle \tau \rangle_{0}\left[Q\right]=1+K_{\mathrm{SV}}\left[Q\right]$$

F_0_ and F are the fluorescence in the absence and presence of quencher, respectively, k_q_ is the bimolecular quenching constant, τ_0_ is the native lifetime of the fluorophore, and [Q] is the concentration of quencher. It is equal to 6 × 10^11^ 1/M × s.

### 3.3. Antiglycation Capacity of V. opulus Fruit Samples

The *V. opulus* fruit samples and reference substances (chlorogenic acid and aminoguanidine) were assessed in vitro for inhibitory effects on end glycation products (AGE) formation through fluorometric detection of fluorescent AGE. Inhibitory activity of extracts and fractions of *V. opulus* dried fruit had a direct linear relationship between concentration and percentage inhibitory activity of AGE formation (Supplementary Figure S4). The inhibitory effects of *V. opulus* fruit samples as well as chlorogenic acid and aminoguanidine (therapic agent for the inhibition of AGE formation) expressed as IC_50_ value is summarized in Table 4. The antiglycation capacity of *V. opulus* fruit samples depended on both the type of sample and the type of sugar used.

PEAF and PEWF with IC_50_ value equal to 32.32 µg/mL and 33.96 µg/mL exhibited the strongest inhibition of AGE formation in the BSA-fructose in vitro model. In addition, PE, PEWF and PEAF demonstrated stronger antiglycation effect than aminoguanidine (IC_50_ = 106.08 µg/mL) and chlorogenic acid (IC_50_ = 58.87 µg/mL). Alternatively, testing the same group of samples for their antiglycation activity in the BSA-glucose model showed aminoguanidine as the strongest inhibitor (IC_50_ = 137.80 µg/mL) followed by PEAF with IC_50_ value equal to 577.50 µg/mL. Antiglycation effects of PE, PEWF and chlorogenic acid in the BSA-glucose assay did not differ significantly (*p* < 0.05)

### 3.4. Antioxidant Capacity of V. opulus Fruit Samples

Antioxidant properties of *V. opulus* samples were estimated by three different methods, as scavenging potential towards stable, synthetic ABTS^•+^ radical cation (ABTS) and towards peroxyl radical (ORAC), and as the potential to reduce ferric to ferrous ion (FRAP). Trolox—a water-soluble analog of vitamin E—was used as an antioxidant standard to determine the Trolox Equivalent (TE). The results for antioxidant capacity of tested samples are presented in Table 5. Significant differences (*p* < 0.05) were found among the analyzed *V. opulus* samples in the antioxidant capacity. The TE values varied from 0.61 to 6.41 mM TE/g of extract or fraction in ABTS assay, from 0.40 to 5.18 mM/g in ORAC assay, and in the range 1.15–24.12 mM/g in FRAP method. The antioxidant capacity of *V. opulus* samples investigated was in the following order: PEAF > PEWF > PE > CE, regardless of the method used.

### 3.5. The Phenolic Compounds Content of V. opulus Fruit Samples

**Figure 3:**
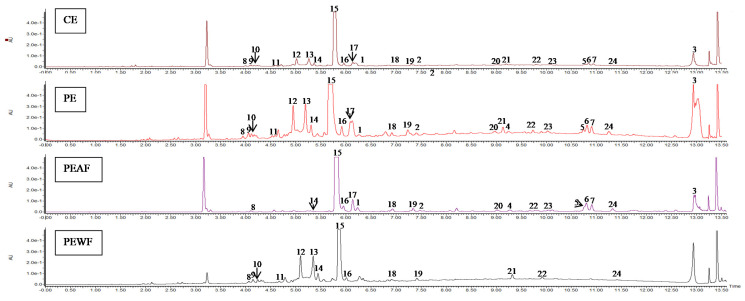
UPLC chromatograms of phenolic compounds in *V. opulus* fruit samples at 280 nm. Refer to Table 7 for the identification of each numbered peak. CE—crude extract, PE—purified extract, PEAF—ethyl acetate fraction of PE, PEWF—water fraction of PE.

The results of quantitative analysis of *V. opulus* fruit extracts (CE, PE) and fractions (PEAF, PEWF) determined by UPLC method are presented in Table 6 and Table 7. Figure 3 presents the chromatograms of *V. opulus* fruit samples at 280 nm.

According to UPLC analysis, the CE, PE and PEAF showed the presence of four groups of phenolic compounds, such as flavanols, flavalignans, hydroxycinnamic acids, and flavonols. For comparison, no flavanols and flavalignans were found in PEWF fraction. On the basis of our results from the UPLC assay concerning low molecular weight phenolics, hydroxycinnamic acids dominated in all samples, while flavonols were the lowest (Table 6). Hydroxycinnamic acids accounted for approximately 84% of the total phenolics in CE, PE and PEAF, and almost 98% of the phenolic compounds determined in PEWF. The purification process on the Sep-Pak C18 column was effective in removing CE non-phenolic compounds as PE was characterized by an approximately 5-fold increase in flavanols and hydroxycinnamic acids, a 6-fold increase in flavonols and proanthocyanidins, and a 7.5-fold increase in flavalignans. As a result of fractionation of the aqueous solution of PE with ethyl acetate, the acetate fraction (PEAF) was 4.7-fold and 4-fold richer in total phenolics and hydroxycinnamic acids, respectively, than the aqueous residue. Moreover, flavanols and flavalignans were only present in PEAF, with the recovery relative to PE being 28.2% and 72.9%, respectively.

In the tested samples, there were 24, 23, 18, and 14 phenolic compounds detected in PE, CE, PEAF and PEWF, respectively (Table 7 and Figure 3). UPLC results revealed that the contents of selected phenolic compounds in different *V. opulus* samples showed significant differences (*p* < 0.05). As the main phenolic compound in all samples, the chlorogenic acid amount in CE was equal to 65.62 mg/g, whereas in PE, PEAF and PEWF it reached 300.23 mg/g, 795.72 mg/g and 152.05 mg/g, respectively. Quantitatively, (epi)-catechin hexoside was the second-most prominent component in all samples with the exception of PEWF in which dicaffeoylquinic acid derivatives were found next after chlorogenic acid.

## 4. Discussion

In Poland, *V. opulus* L. is mainly cultivated as an ornamental shrub and its red and shiny fruits are rarely used as food. However, in Turkey, Russia and Ukraine, these fruits are used in traditional cuisine. *V. opulus* fruits are widely used in natural medicine for the relief of asthma, cold, fever, cough, nervousness, rheumatoid diseases, menstrual cramps, uterine and urinary tract infections, and to treat high blood pressure [36]. Our previous studies conducted both in cell-based and cell-free models, revealed that the compounds included in the *V. opulus* fresh fruits exhibited antidiabetic properties [33,34,35]. Contrary to the previous studies, we chose dried fruits as the research material due to their commercial availability. The present research is in line with the trend of searching safe and effective antidiabetic agents from natural sources.

This is the first study of the effect of *V. opulus* dried fruit extracts on both the activity of carbohydrate digestive enzymes and the process of AGE formation. Inhibition of α-glucosidase and α-amylase can prevent the postprandial surge of glucose and help to maintain its safe level in blood which is one of the primary targets for the treatment and management of the early stage of T2DM [47]. Additionally, inhibition of AGE formation is another approach in delaying or preventing the onset of diabetic complications [48]. Many studies conducted on fruits indicated that phenolic compounds were the functional components that played a major role in their hypoglycemic effect [7,8,9,12,27,31,49,50,51,52]. Thus, in order to verify this hypothesis in our research, the *V. opulus* fruit samples varying in terms of degree of purification of phenolic compounds and thus in the presence of other non-phenolic components were used. In this study four samples were obtained from *V. opulus* dried fruits. CE was prepared using 70% acetone as a solvent, PE was obtained from CE by solid phase extraction method as a purification step, and next PE was fractionated into ethyl acetate and water phase (PEAF and PEWF). Two analyzed *V. opulus* fruit extracts were characterized by different content of phenolic compounds, from 87.42 mg/g of CE to 418.39 mg/g of PE (Table 6). Moreover, as a result of PE fractionation by the liquid–liquid extraction method, PEAF (the ethyl acetate fraction) contained as much as 977.08 mg of total phenolics per gram and PEWF (the water fraction) with 206.87 mg/g of fraction. In addition, PE was the most abundant in proanthocyanidins, almost 95% converted to PEWF, which may indicate the presence of proanthocyanidins with a high degree of polymerization. According to Saucier et al. [53] the proanthocyanidin oligomers are soluble in ethyl acetate while the polymers remain in the aqueous phase. The presence of tannins in the form of polymers was also confirmed by the absence of peaks corresponding to the flavanols in the UPLC chromatograms of PEAF (Figure 3 and Table 7). According to Perova et al. [54], proanthocyanidins are a quantitatively significant component of the fresh *V. opulus* fruits with content 201–528 mg/100 g fresh weight and they accounted over 49.9% of total phenolics. However, UPLC results showed hydroxycinnamic acids dominated in all *V. opulus* samples tested. This group of simple phenolic compounds constituted over 84% of the total phenolics in the analyzed samples, with the highest participation (over 97%) in PEWF. Chlorogenic acid was identified as the major phenolic compound in all *V. opulus* fruit samples with amount 65.62 mg/g of CE, whereas in PE, PEAF and PEWF it reached 300.23 mg/g, 795.72 mg/g, and 152.05 mg/g, respectively. Chlorogenic acid has also been indicated as the main phenolic compound of *V. opulus* fruit by other authors [46,55,56,57].

According to our results, the composition of phenolic compounds of *V. opulus* fruit samples had a significant impact on both the activity of α-amylase and α-glucosidase, the process of AGE formation, as well as the antioxidant activity of the analyzed *V. opulus* fruit samples. In this study, the porcine pancreatic α-amylase and rat intestinal α-glucosidase models have been used because these enzymes play a crucial role in starch breakdown during digestion and glucose uptake. Pancreatic α-amylase is an endoglucosidase presents in pancreatic juice secreted into the intestinal lumen [7] and it digests the starch to maltose, maltotriose and dextrins, which are then broken down into glucose by other carbohydrases, e.g., α-glucosidase [19]. α-Glucosidase is a specific membrane-bound enzyme in the small intestine catalyzing the hydrolytic cleavage of disaccharides into monosaccharides [58]. In our study, crude extract (CE) showed the lowest inhibitory potential against carbohydrate digestive enzymes, regardless of both enzyme and substrate types (Table 1). Removal of non-phenolic compounds from CE by the solid phase extraction method increased the inhibitory activity of PE on average 5-fold in the case of α-amylase and 9-fold in the case of α-glucosidase. The most active inhibitor of α-amylase was PEWF while PEAF exhibited the highest inhibitory activity against α-glucosidase. The reference of the obtained results to the phenolic composition of these fractions suggests that proanthocyanidin polymers, which remain in the aqueous phase, are mainly responsible for the anti-amylase activity of *V. opulus* fruits, and hydroxycinnamic acids, as well as flavanol oligomers and flavalignans for α-glucosidase inhibition. PEWF contained 2.6-fold more proanthocyanidins, but 4-fold less hydroxycinnamic acids and 1.7-fold less flavonols than PEAF (Table 6). Proanthocyanidins from grape seed and skin, acacia bark, persimmon peel and leaf, apple, and almond peel skin have been previously reported to inhibit α-amylase [59]. Additionally, proanthocyanidins with a high degree of polymerization compared to those with low degree had a greater effect on α-amylase activity in mice [60]. The higher activity of PEWF than PEAF on α-amylase may be due not only to the presence of proanthocyanidins but also to dicaffeoylquinic acids which were not generally present in PEAF. Narita and Inouye [61] showed that the decreasing rank of hydroxycinnamic acid derivatives on porcine pancreas α-amylase isozyme I was as follows: dicaffeoylquinic acids > caffeoylquinic acids > feruoylquinic acids. Our results demonstrated a very weak inhibitory activity of chlorogenic acid towards α-amylase (the highest IC_50_ values, on average 2.7-fold and 14-fold higher than the IC_50_ values for CE and PE, respectively).

The dominant role of hydroxycinnamic acids, including chlorogenic acid in inhibition of α-glucosidase is confirmed by similar IC_50_ values for chlorogenic acid and PEAF—the most active *V. opulus* sample (Table 1). Oki et al. [62] demonstrated that the activity of rat α-glucosidase depends on disaccharide type. Thus, both maltose (a breakdown product of starch) and sucrose (the major disaccharide in food) were used as substrates for rat intestine α-glucosidase in the test systems used. The IC_50_ values of all *V. opulus* fruit samples and acarbose (a well-known and standard antidiabetic drug) were lower in the maltose-containing system. These differences are due to the different affinity of the enzyme for the substrate as demonstrated by Oki et al. [62] because maltose had near 8-fold higher affinity for rat α-glucosidase than sucrose (Michaelis constants were equal to 3.0 and 22.8 mM, respectively). On the other hand, the inhibitory activity of chlorogenic acid (the dominant phenolic compound in *V. opulus* samples) was comparable in both assay systems. Study of Iwai and co-workers [31] confirmed inhibitory activity of chlorogenic acid against sucrase and maltase from rat small intestine with IC_50_ equal to 0.50 and 8.81 mg/mL, respectively. This study also showed weaker inhibitory activity of this phenolic acid on α-amylase porcine pancreas (IC_50_ = 13.17 mg/mL) which is in line with our results. However, Pollini et al. [26] demonstrated that chlorogenic acid had higher inhibitory activity against α-amylase than other phenolics acids (e.g., caffeic acid, *p*-coumaric acid, sinapic acid, and syringic acid) and 5-fold lower activity compared to acarbose. In addition, the inhibitory activities of the *V. opulus* fruit samples against both α-amylase and α-glucosidase were lower than the acarbose, probably due to the complexity of the chemical composition of fruit matrices, and weak inhibitory activity of chlorogenic acid—quantitatively the main component of the tested samples.

The evaluation of the inhibition mode showed that PE and PEWF (the most active *V. opulus* samples against α-amylase) and acarbose inhibited the α-amylase uncompetitively (Supplementary Figure S2, Table 2). In this case, inhibitor binds only to the complex form between the enzyme and the substrate, i.e., potato or rice starch. On the other hand, PE and PEAF (the most active *V. opulus* samples against α-glucosidase) as well as acarbose displayed mixed mode of inhibition towards α-glucosidase (Supplementary Figure S3, Table 2). This means that the inhibitor may bind to the enzyme whether or not the enzyme has already bound the substrate, but has a greater affinity for one state or the other. Contrary, the Lineweaver–Burk plots showed that chlorogenic acid inhibits α-amylase noncompetitively and has a competitive mode towards α-glucosidase. Karim et al. [63] and Sun et al. [64] reported that chlorogenic acid has a mixed type inhibitory effect on porcine pancreatic α-amylase hydrolysis of potato and maize starch, respectively. In line with our results, a chlorogenic-rich fraction from *Smilax aristolochiifolia* root inhibited α-amylase activity in an uncompetitive manner and α-glucosidase in a noncompetitive mode [65]. To the best of our knowledge, the inhibitory mode of *V. opulus* fruit extracts and fractions are reported for the first time.

Interactions between both enzymes and *V. opulus* fruit components were also confirmed by fluorescence spectroscopy for PE. Under our measurement condition, PE reduced the fluorescence intensity of α-amylase and α-glucosidase, and the maximal absorption peak was slightly red-shifted. The same effects were found with the addition of chlorogenic acid and its derivatives to the α-glucosidase from *Saccharomyces cerevisiae* [66]. The authors suggested that these changes led to a polarity variation for tryptophan (Trp) residues in the enzyme and made the microenvironment change from hydrophilic to hydrophobic, which resulted in greater exposure of Trp residues and development of the protein structure. In the present study, the value of the bimolecular quenching constant is equal to 6 × 10^11^ 1/M × s, and is above the diffusion-limited rate constant of the biomolecule equal to 1 × 10^10^ 1/M × s [67]. It suggests the involvement of static mechanism in the fluorescence quenching of the α-amylase and α-glucosidase with PE. Thus, the binding affinity of PE to the studied enzymes may be estimated by the determined Stern–Volmer constants.

Chronic hyperglycemia promotes the formation of free radicals, that further increases the formation of reactive oxygen species, which in turn lead to a state of oxidative stress—a key factor in the development of diabetic complications [48]. There are increasing evidence that phenolic compounds due the antioxiadant properties may protect cell constituents against oxidative damage and, therefore, limit the risk of various degenerative diseases associated with oxidative stress [68,69]. In the current study, all *V. opulus* fruit samples demonstrated antioxidant activity, indicated by scavenging potential towards stable, synthetic ABTS^•+^ radical cation (ABTS) and towards peroxyl radical (ORAC), and as Fe^3+^ reducing activity (FRAP). The antioxidant capacity of these samples followed this order: PEAF > PEWF > PE > CE, regardless of the method (Table 5). Chlorogenic acids, including mono- and dicaffeoylquinic acids as well as coumaroylquinic and feruoylquinic acid which were identified in *V. opulus* fruit samples by UPLC method (Table 7) are known to be potent antioxidants [70]. Dicaffeoylquinic acids possessed better antioxidant activities, mostly because they have more hydroxyl groups than caffeoylquinic acids. On the other hand, caffeoylquinic acid isomers showed quite similar antioxidant activities, indicating that the position of esterification on the quinic moiety of caffeoylquinic acid had no effect on its antioxidant activities.

At the early stage of T2DM, there is a relationship between hyperglycemia, increased oxidative stress, and excessive AGE formation [48]. The prevention of protein glycation is a viable strategy to prevent AGE formation since these molecules have ability to modify the chemical and functional properties of various biological structures [71]. All *V. opulus* fruit samples (extracts and fractions), as well as chlorogenic acid, showed inhibitory activity against AGE formation and had significantly (*p* < 0.05) lower IC_50_ values than the positive control, aminoguanidine in the BSA-glucose model (Table 4). Other relationships were found in the BSA-fructose measurement system, where only crude extract (CE) of *V. opulus* fruit was a weaker inhibitor of AGE formation compared to aminoguanidine. The higher IC_50_ values obtained with BSA-glucose compared to BSA-fructose are probably due to higher reactivity of ketose sugar than which has a more open-chain structure than aldose sugar [51]. Regardless of the test used, the ethyl acetate fraction (PEAF) showed the strongest inhibitory properties against the formation of fluorescent AGE. In studies by Justino et al. [52] ethyl acetate fraction from both ethanol and aqueous crude extracts of *Eugenia dysenterica* fruit pulp also showed the lowest IC_50_ values in the BSA-fructose model. This fraction in both studies was characterized by the highest concentrations of phenolic compounds. The ethyl acetate fraction of *V. opulus* fruit contained different groups of phenolics with a predominance of chlorogenic acid and much less flavanols and flavalignans. This fraction of *E. dysenterica* fruit was composed of various phenolic compounds such as ferulic acid derivatives, flavonols and flavanols. These results may indicate that simple phenolic acids as well as more complex flavonoids can be able to inhibit the AGEs formation. In addition, some studies showed antiglycation activity of *V. opulus* fruit components such as 3,5-dicaffeoylquinic acid [72], chlorogenic and caffeic acid [73,74], catechins and procyanidins [75]. The antiglycation potential of *V. opulus* fruit had not been previously studied.

## 5. Conclusions

The potential ability of *V. opulus* dried fruit extracts, especially enriched with phenolic compounds to inhibit α-amylase and α-glucosidase, and AGE formation, and exhibition of antioxidative activities could be some of antidiabetic properties. The effects listed above are likely attributed to fruit phenolic compounds. The purification of crude extract resulted in extract enriched with phenolic compounds showing higher inhibitory activity against carbohydrate hydrolases and AGE formation, as well as antioxidant capacity. Further separation of the aqueous PE solution with ethyl acetate led to the obtaining of the acetate fraction rich in chlorogenic acid, proanthocyanidin oligomers and flavalignans with the highest inhibitory activity against α-glucosidase, formation of glycation products and antioxidant potential. The highest inhibitory activity of the water fraction of PE against α-amylase is related to proanthocyanidin polymers and chlorogenic acids, especially dicaffeoylquinic acids. This study suggested that the *V. opulus* fruits can serve as prospective source of natural phenolics, which could be developed as potential effective component of nutraceuticals and functional foods, especially after isolating a bioactive fraction, e.g., with ethyl acetate. The present results broaden knowledge regarding phytochemical and biological potential of non-conventional fruits, especially those related to the inhibition of AGE formation and enzymes activity that hydrolyze carbohydrates.

## Figures and Tables

**Table 1:** IC_50_ values (µg/mL) of the *V. opulus* fruit samples and reference substances (chlorogenic acid and acarbose) for the inhibition of α-amylase and α-glucosidase in the presence of different substrates.

Sample	α-Amylase		α-Glucosidase	
	Substrate			
	Potato Starch	Starch from Rice	Maltose	Sucrose
CE	400.70 ± 8.29d	572.12 ± 19.41d	1922.83 ± 77.14e	3244.88 ± 111.28d
PE	61.51 ± 0.72b	132.42 ± 3.33b	180.09 ± 4.67cd	447.60 ± 29.22c
PEAF	378.71 ± 3.64c	509.22 ± 1.81c	61.87 ± 1.97ab	110.06 ± 1.12b
PEWF	24.17 ± 0.23a	124.90 ± 2.70b	253.71 ± 6.79d	324.93 ± 11.32c
Chlorogenic acid	833.61 ± 16.34e	1963.57 ± 30.49e	103.37 ± 5.29bc	99.81 ± 0.75ab
Acarbose	13.33 ± 0.17a	30.57 ± 1.19a	0.051 ± 0.001a	1.37 ± 0.04a

The values within a same column with different letters are significantly different (*p* < 0.05).

**Table 2:** Related parameter on the inhibitory kinetics of the highest concentration of *V. opulus* samples, chlorogenic acid and acarbose on α-amylase and α-glucosidase.

Enzyme	Substrate	Sample	Concentration (μg/mL)	K_m_ (μg/mL)	V_max_ (OD/min)	K_i_ (μg/mL)	Inhibition Type
α-amylase	Potato starch	Control PEWF	- 25.64	1.25 0.32	1.32 0.40	/ 11.14	uncompetitive
		Control PE	- 54.95	1.00 0.21	1.10 0.24	/ 15.22	uncompetitive
		Control Chlorogenic acid	- 769.23	0.59 0.83	0.80 0.80	/ 1225.78	competitive
		Control Acarbose	- 6.62	1.43 0.20	1.41 0.22	/ 1.24	uncompetitive
	Rice starch	Control PEWF	- 153.86	1.25 0.18	0.63 0.08	/ 20.82	uncompetitive
		Control PE	- 153.86	1.00 0.15	0.51 0.07	/ 22.84	uncompetitive
		Control Chlorogenic acid	- 1923.1	1.67 3.33	0.60 0.60	/ 1886.20	competitive
		Control Acarbose	- 19.86	2.50 0.19	1.27 0.10	/ 1.78	uncompetitive
α-glucosidase	Maltose	Control PEAF	- 61.73	0.40 0.63	0.021 0.014	/ 46.44	mixed
		Control PE	- 185.19	0.18 0.69	0.023 0.013	/ 89.25	mixed
		Control Chlorogenic acid	- 191.31	0.40 0.40	0.021 0.009	/ 145.41	noncompetitive
		Control Acarbose	- 0.05	0.40 0.69	0.021 0.012	/ 0.021	mixed
	Sucrose	Control PEAF	- 111.11	6.67 9.09	0.006 0.005	/ 159.10	mixed
		Control PE	- 476.19	5.56 6.67	0.008 0.004	/ 379.75	mixed
		Control Chlorogenic acid	- 191.31	7.69 7.69	0.005 0.003	/ 181.21	noncompetitive
		Control Acarbose	- 1.35	6.67 8.33	0.006 0.004	/ 1.52	mixed

“/”—represents no inhibition constants (Ki) value.

**Table 3:** The changes in fluorescence lifetimes (τ_1_ [ns], τ_2_ [ns], τ_3_ [ns], <τ> [ns]) and their fractional fluorescence of the tested enzymes and their changes upon addition of PE to the final concentration 1.36 × 10^−4^ M.

Sample	τ_1_ [ns]	f_1_ [%]	τ_2_ [ns]	f_2_ [%]	τ_3_ [ns]	f_3_ [%]	<τ> * [ns]	χ2
α-Amylase	0.89 ± 0.04	13.73	3.13 ± 0.05	58.35	8.90 ± 0.11	27.92	12.40398	1.169
α-Amylase + PE	0.84 ± 0.04	14.18	3.10 ± 0.05	58.66	8.83 ± 0.11	27.17	12.12822	1.210
α-Glucosidase	0.62 ± 0.03	11.51	3.02 ± 0.04	62.61	7.02 ± 0.11	25.88	11.00854	1.196
α-Glucosidase + PE	0.66 ± 0.03	12.29	3.12 ± 0.05	64.20	7.19 ± 0.13	23.51	11.50588	1.028

* Defined as <τ> = Σf_i_·τ_i_; PE concentration = 1.36 × 10^−4^ M chlorogenic acid equivalents.

**Table 4:** IC_50_ values (µg/mL) of the *V. opulus* fruit samples and reference substances (chlorogenic acid and aminoguanidine) for the inhibition of AGE formation.

Sample	BSA-Fructose Model	BSA-Glucose Model
CE	182.03 ± 2.52e	4966.22 ± 110.32d
PE	40.78 ± 0.02b	895.33 ± 9.39c
PEAF	32.32 ± 0.28a	577.50 ± 5.32b
PEWF	33.96 ± 0.27a	803.27 ± 4.79c
Chlorogenic Acid	58.87 ± 0.93c	869.94 ± 6.25c
Aminoguanidine	106.08 ± 3.17d	137.80 ± 3.44a

Values that are followed by different letters within each column are significantly different (*p* < 0.05).

**Table 5:** Antioxidant capacity of the *V. opulus* fruit samples expressed as Trolox equivalents (mM TE/g of sample).

Sample	ABTS	FRAP	ORAC
CE	0.61 ± 0.02a	0.40 ± 0.01a	1.15 ± 0.03a
PE	2.24 ± 0.03b	1.80 ± 0.08b	3.33 ± 0.10b
PEAF	6.41 ± 0.25d	5.18 ± 0.06c	24.12 ± 0.91d
PEWF	5.13 ± 0.08c	5.02 ± 0.15c	10.70 ± 0.40c

Values that are followed by different letters within each column are significantly different (*p* < 0.05).

**Table 6:** The content (mg/g of extract or fraction) of phenolic compounds of *V. opulus* fruit samples.

Phenolic Compounds	*V. opulus* fruit Sample			
	CE	PE	PEAF	PEWF
Flavanols ^1^	11.53 ± 0.07a	52.63 ± 1.76b	98.50 ± 3.90c	-
Flavalignans ^1^	1.13 ± 0.04a	8.42 ± 0.20b	40.70 ± 0.27c	-
Hydroxycinnamic acids ^1^	73.90 ± 0.51a	352.26 ± 3.57c	829.26 ± 0.51d	201.81 ± 0.63b
Flavonols ^1^	0.86 ± 0.01a	5.08 ± 0.12b	8.62 ± 0.17c	5.06 ± 0.05b
Total phenolics ^1^	87.42 ± 0.63a	418.39 ± 5.65c	977.08 ± 4.85d	206.87 ± 0.68b
Total proanthocyanidins ^2^	12.26 ± 0.46a	74.72 ± 7.90d	21.96 ± 1.35b	57.33 ± 1.85c

“-”—not identified; ^1^ determined by UPLC analysis; ^2^ determined by spectrophotometric method; The values within a same raw with different letters are significantly different (*p* < 0.05).

**Table 7:** The phenolic compounds content in *V. opulus* fruit samples.

Peak	Phenolic Compound	CE	PE	PEAF	PEWF
		mg/g of Extract		mg/g of Fraction	
Flavanols					
1	Procyanidin tetramer ^a^	0.23 ± 0.00a	1.81 ± 0.03c	1.28 ± 0.04b	-
2	Procyanidin C1	0.80 ± 0.00a	2.03 ± 0.11c	0.99 ± 0.03b	-
3	(Epi)-catechin hexoside ^b^	10.50 ± 0.07a	48.79 ± 1.62b	96.23 ± 3.83c	-
Flavalignans					
4	Cinchonain Ix ^c^	-	0.72 ± 0.02a	3.88 ± 0.02b	-
5	Cinchonain Ix ^c^	0.19 ± 0.03a	1.90 ± 0.06b	23.13 ± 0.21c	-
6	Cinchonain Ix ^c^	0.54 ± 0.01a	3.23 ± 0.07b	11.55 ± 0.04c	-
7	Cinchonain Ix ^c^	0.40 ± 0.00a	2.57 ± 0.05c	2.14 ± 0.00b	-
Hydroxycinnamic acids					
8	Neochlorogenic acid	0.20 ± 0.00a	1.41 ± 0.05b	1.97 ± 0.03c	1.39 ± 0.00b
9	Dicaffeolquinic acid I ^d^	0.20 ± 0.00a	1.69 ± 0.08b	-	1.66 ± 0.00b
10	Dicaffeolquinic acid II ^d^	0.09 ± 0.00a	1.38 ± 0.05b	-	1.58 ± 0.00c
11	Dicaffeolquinic acid III ^d^	0.23 ± 0.00a	0.55 ± 0.02b	-	1.22 ± 0.00c
12	Dicaffeolquinic acid IV ^d^	2.39 ± 0.01a	15.14 ± 0.47b	-	17.35 ± 0.01c
13	Dicaffeolquinic acid V ^d^	2.92 ± 0.00a	20.04 ± 0.20c	-	19.68 ± 0.04b
14	Dicaffeolquinic acid VI ^d^	0.68 ± 0.00b	4.37 ± 0.12c	0.37 ± 0.00a	4.40 ± 0.01c
15	Chlorogenic acid	65.62 ± 0.49a	300.23 ± 2.33c	795.72 ± 0.03d	152.05 ± 0.52b
16	Cryptochlorogenic acid	0.40 ± 0.00a	1.81 ± 0.05c	4.78 ± 0.01d	0.97 ± 0.01b
17	Caffeic acid	0.38 ± 0.01a	1.67 ± 0.02b	8.37 ± 0.11c	-
18	Caffeoylquinic acid ^d^	0.10 ± 0.00a	0.66 ± 0.06b	3.84 ± 0.24c	0.40 ± 0.02ab
19	Coumaroylquinic acid ^d^	0.46 ± 0.00a	2.48 ± 0.09c	9.99 ± 0.01d	1.11 ± 0.02b
20	Feruoylquinic acid ^d^	0.23 ± 0.00a	0.83 ± 0.03b	4.22 ± 0.08c	-
Flavonols					
21	Quercetin 3-sambubioside ^e^	0.27 ± 0.00a	1.35 ± 0.04b	-	2.01 ± 0.00c
22	Quercetin 3-rutinoside	0.41 ± 0.00a	2.22 ± 0.02c	1.70 ± 0.15b	2.80 ± 0.03d
23	Quercetin 3-glucoside	0.07 ± 0.01a	0.46 ± 0.03b	2.05 ± 0.01c	-
24	Quercetin 3-rhamnoside	0.11 ± 0.00a	1.05 ± 0.03c	4.87 ± 0.01d	0.25 ± 0.02b

“-”—not identified. The content expressed as equivalents of: ^a^—procyanidin C1, ^b^—(+)-catechin, ^c^—cinchonine, ^d^—chlorogenic acid, ^e^—quercetin 3-glucoside. The means within a same raw with different letters are significantly different.

## Data Availability

The data presented in this study are available on request from the corresponding author.

## Supplementary Materials

The following are available online at https://www.mdpi.com/article/10.3390/antiox10060989/s1. Figure S1: Inhibitory effects of *V. opulus* fruit samples on α-amylase activity in the presence of potato starch (A) and starch from rice (B), and on α-glucosidase activity in the presence of maltose (C) and sucrose (D), Figure S2: Inhibition mode of the *V. opulus* fruit samples (PE and PEWF) and reference substance (chlorogenic acid and acarbose) on α-amylase in the presence of potato starch (A) and starch from rice (B), Figure S3: Inhibition mode of the *V. opulus* fruit samples (PE and PEAF) and reference substance (chlorogenic acid and acarbose) on α-glucosidase in the presence of maltose (A) and sucrose (B), Figure S4: The inhibitory effects of *V. opulus* fruit samples on advanced glycation-end products (AGE) formation in BSA-fructose (A) and BSA-glucose (B) models.
