# Supplementary material for: Glycoside Hydrolases and Non-Enzymatic Glycation Inhibitory Potential of Viburnum opulus L. Fruit—In Vitro Studies
Source: Antioxidants (Basel). 2021 Jun 21;10(6):989. doi: 10.3390/antiox10060989 (PMC8235151; doi:10.3390/antiox10060989)
Supplement: Supplementary file 1 [file antioxidants-10-00989-s001.zip › antioxidants-1255306-supplementary.pdf]

SUPPLEMNETARY MATERIALS

Glycoside Hydrolases and Non-Enzymatic Glycation Inhibitory Potential of *Viburnum opulus* L. Fruit—In Vitro Studies

Dominika Kajszczak [1,*], Agnieszka Kowalska-Baron [2] and Anna Podsędek [1]

1 Institute of Molecular and Industrial Biotechnology, Faculty of Biotechnology and Food Sciences, Lodz University of Technology, Stefanowskiego 2/22, 90-537 Łódź, Poland; anna.podsedek@p.lodz.pl
2 Institute of Natural Products and Cosmetics, Faculty of Biotechnology and Food Sciences, Lodz University of Technology, Stefanowskiego 2/22, 90-537 Łódź, Poland; agnieszka.kowalska-baron@p.lodz.pl;

* Correspondence: dominika.kajszczak@dokt.p.lodz.pl

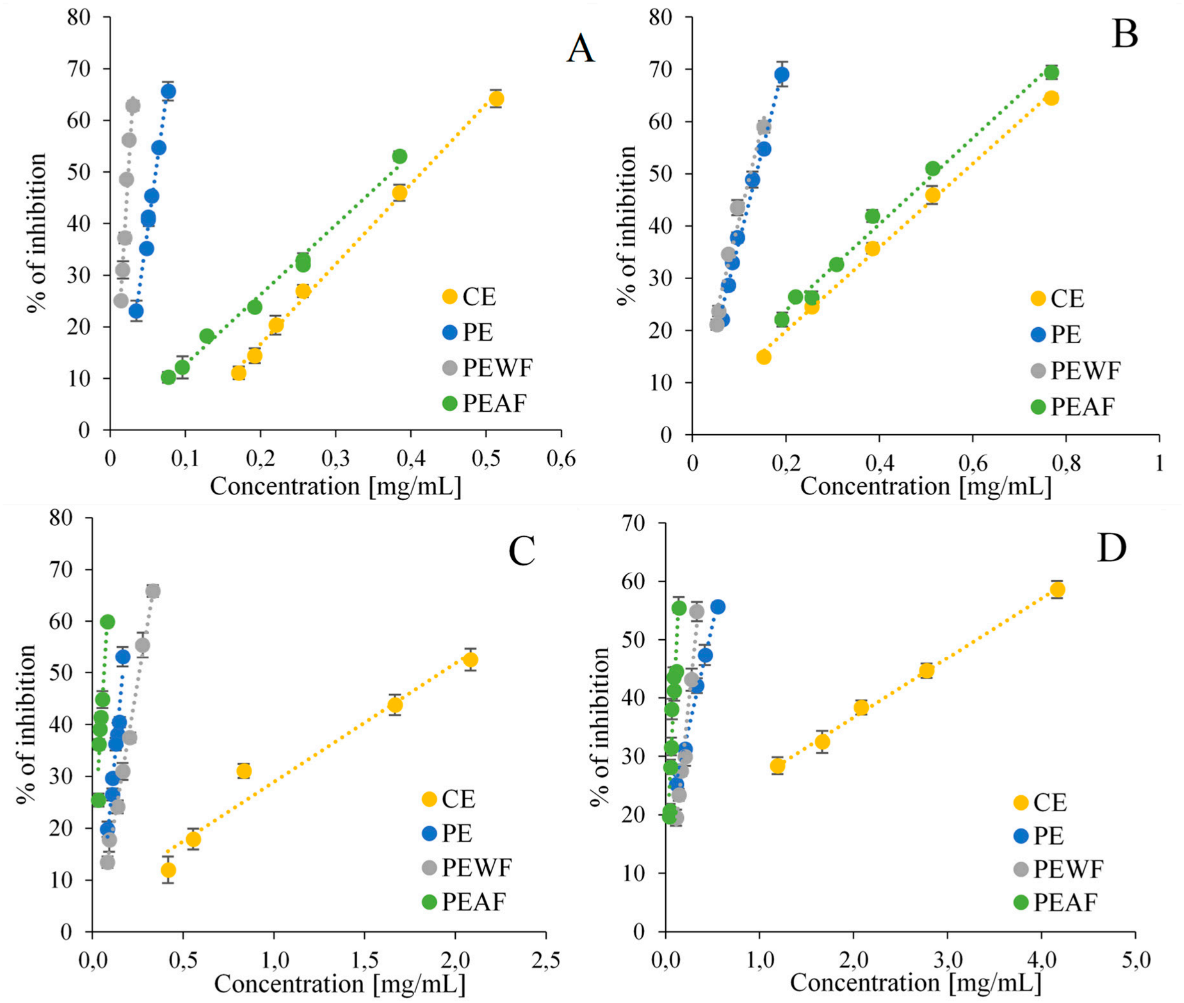

Figure S1. Inhibitory effects of *V. opulus* fruit samples on α-amylase activity in the presence of potato starch (**A**) and starch from rice (**B**), and on α-glucosidase activity in the presence of maltose (**C**) and sucrose (**D**). CE—crude extract, PE—purified extract, PEAF—ethyl acetate fraction of PE, PEWF—water fraction of PE.

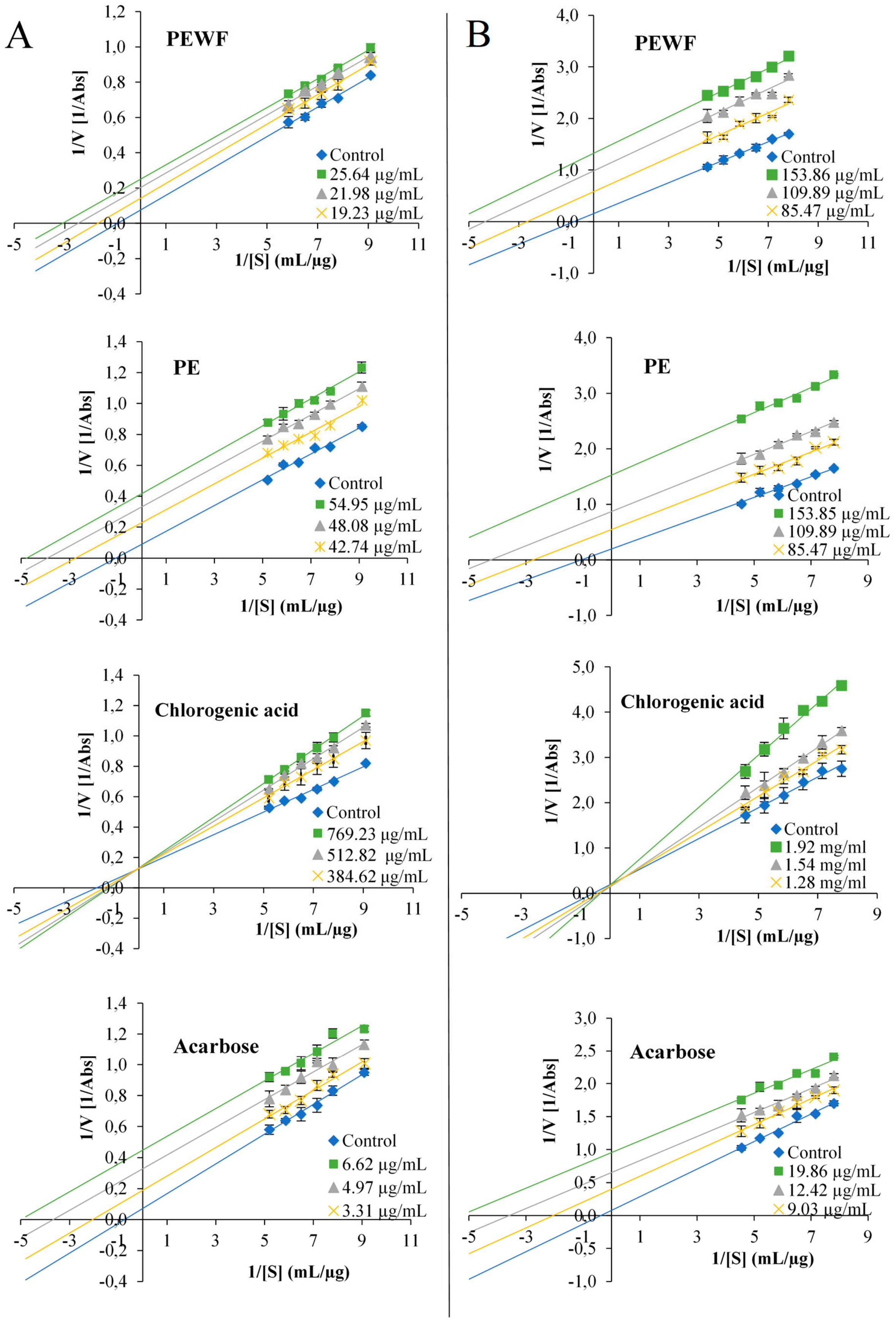

Figure S2. Inhibition mode of the *V. opulus* fruit samples (PE and PEWF) and reference substance (chlorogenic acid and acarbose) on α-amylase in the presence of potato starch (**A**) and starch from rice (**B**).

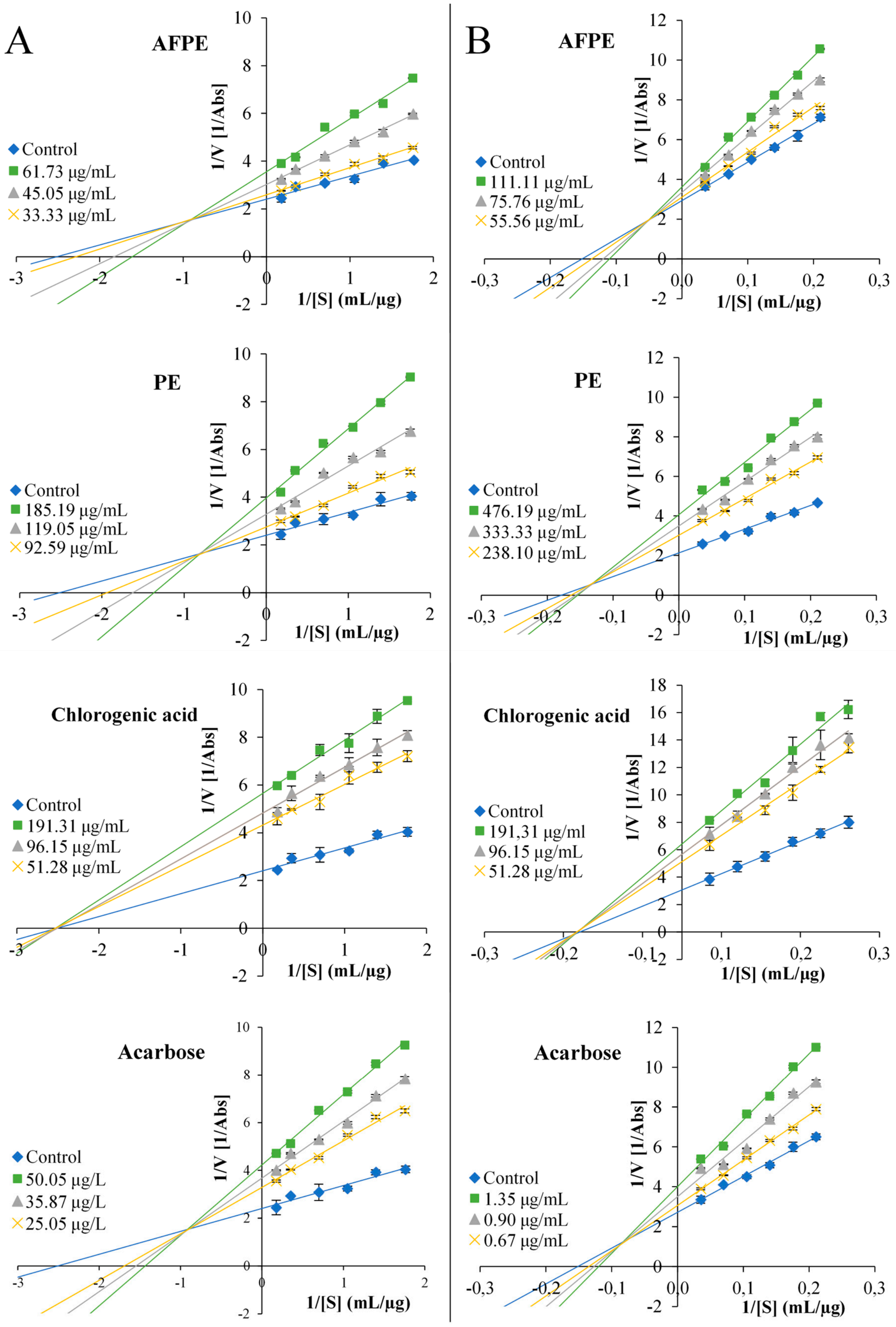

Figure S3. Inhibition mode of the *V. opulus* fruit samples (PE and PEAF) and reference substance (chlorogenic acid and acarbose) on α-glucosidase in the presence of maltose (**A**) and sucrose (**B**).

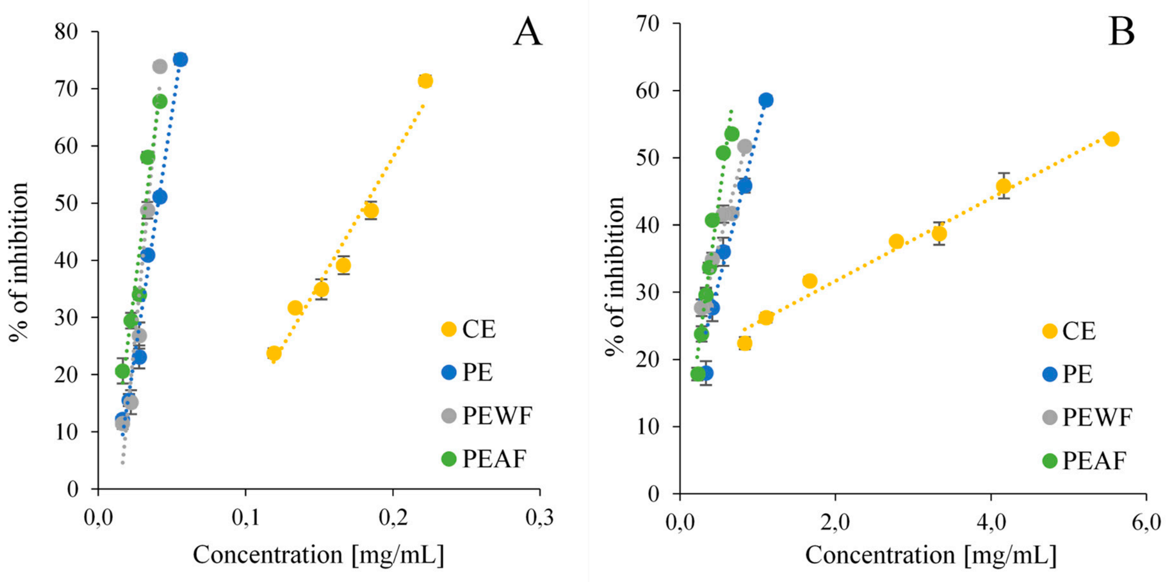

Figure S4. The inhibitory effects of *V. opulus* fruit samples on advanced glycation-end products (AGE) formation in BSA-fructose (**A**) and BSA-glucose (**B**) models. CE—crude extract, PE—purified extract, PEAF—ethyl acetate fraction of PE, PEWF—water fraction of PE.
